# Transcriptional Activation of the *mrkA* Promoter of the *Klebsiella pneumoniae* Type 3 Fimbrial Operon by the c-di-GMP-Dependent MrkH Protein

**DOI:** 10.1371/journal.pone.0079038

**Published:** 2013-11-14

**Authors:** Ji Yang, Jonathan J. Wilksch, Jason W. H. Tan, Dianna M. Hocking, Chaille T. Webb, Trevor Lithgow, Roy M. Robins-Browne, Richard A. Strugnell

**Affiliations:** 1 Department of Microbiology and Immunology, The University of Melbourne, Parkville, Victoria, Australia; 2 Department of Microbiology, Monash University, Clayton, Victoria, Australia; East Carolina University School of Medicine, United States of America

## Abstract

The Gram-negative bacterial pathogen *Klebsiella pneumoniae* forms biofilms to facilitate colonization of biotic and abiotic surfaces. The formation of biofilms by *K. pneumoniae* requires the expression of type 3 fimbriae: elongate proteinaceous filaments extruded by a chaperone-usher system in the bacterial outer membrane. The expression of the *mrkABCDF* cluster that encodes this fimbrial system is strongly positively regulated by MrkH, a transcriptional activator that responds to the second messenger, c-di-GMP. In this study, we analyzed the mechanism by which the MrkH protein activates transcriptional initiation from the *mrkA* promoter. A mutational analysis supported by electrophoretic mobility shift assays demonstrated that a 12-bp palindromic sequence (the MrkH box) centered at −78.5 is the binding site of MrkH. Deletion of half a turn, but not a full turn, of DNA located between the MrkH box and the *mrkA* promoter destroyed the ability of MrkH to activate *mrkA* transcription. In addition, a 10-bp AT-rich sequence (the UP element) centered at −63.5 contributed significantly to MrkH-dependent *mrkA* transcription. *In vivo* analysis of *rpoA* mutants showed that the R265 and E273 determinants in the C-terminal domain of RNA polymerase α subunit are needed for MrkH-mediated activation of *mrkA* transcription. Furthermore, results from mutagenesis of the *mrkH* gene suggest that the N-terminal region of the protein is involved in transcriptional activation. Taken together, our results suggest that MrkH activates *mrkA* expression by interacting directly with RNA polymerase, to overcome the inefficient transcriptional initiation caused by the presence of defective core promoter elements.

## Introduction

*Klebsiella pneumoniae* is an opportunistic Gram-negative bacterial pathogen that frequently causes outbreaks of nosocomial pneumonia, catheter-associated urinary tract infections and bacteremia [1], [2], [3], [4], [5], [6]. *K. pneumoniae* is able to form robust biofilms which are required for bacterial colonization on indwelling medical devices [7], [8], [9]. As with numerous other bacteria, biofilm formation of *K. pneumoniae* requires transduction of chemical signals within the bacterial cells and coordinated transcriptional regulation of the genes involved [10], [11], [12]. An important physiological change that occurs within bacterial cells during the conversion from planktonic to biofilm life-styles is the enhancement of levels of the second messenger cyclic di-guanosine monophosphate (c-di-GMP) [13], [14], [15]. Recent studies revealed that c-di-GMP is able to directly modulate the activities of a number of transcriptional regulators that control the expression of genes involved in biofilm formation [16], [17], [18], [19].

*K. pneumoniae* isolates commonly express two well-characterised fimbrial adhesins, type 1 and type 3 fimbriae [20]. The type 1 fimbriae, which are regulated via phase regulation, have been implicated in promoting *K. pneumoniae* colonization and biofilm formation [9], [21]. Type 3 fimbriae have been shown to mediate the initiation of biofilm formation on biotic and abiotic surfaces, as well as being required for mature biofilm development [7], [8], [22], [23], [24]. The various components of type 3 fimbriae are encoded by the *mrkABCDF* operon [25], which is under the transcriptional control by a single σ^70^-dependent promoter located 204 bp upstream from the *mrkA* major fimbrial subunit gene [10], [12]. We have previously shown that transcription from the *mrkA* promoter is highly up-regulated by MrkH, which exerts 49- and 220-fold activation on transcription of the *mrkABCDF* operon in the haploid and multicopy *mrkH* background, respectively [10]. MrkH is encoded within a three-locus cluster (*mrkH-mrkI-mrkJ*) that is located immediately adjacent to the *mrkABCDF* operon [10]. The *mrkI* gene encodes a putative regulatory protein that contains a LuxR-like DNA binding domain and is implicated in type 3 fimbriae regulation [10], [11]. The *mrkJ* gene encodes a phosphodiesterase (PDE) that degrades c-di-GMP and functions as a negative regulator of type 3 fimbriae expression and biofilm formation [10], [26].

MrkH is a novel transcriptional activator that contains a putative c-di-GMP binding site, referred to as a PilZ domain [10], [11]. PilZ domain effector proteins that bind c-di-GMP have been identified in numerous bacteria to relay signals to regulate cellular processes such as motility, exopolysaccharide synthesis and biofilm formation [27]. The PilZ domain contains a characteristic short, flexible loop, which undergoes a conformational change upon c-di-GMP binding [13]. The PilZ family of c-di-GMP-binding proteins includes BcsA, the catalytic α-subunit of cellulose synthase first described in *Gluconacetobacter xylinus* [28], [29]; YcgR, a component of the flagellar machinery found to regulate motility in *E. coli* [30], [31]; and Alg44, a trans-membrane protein that regulates alginate export in *Pseudomonas aeruginosa* [32]. The BcsA-type and YcgR-type proteins are widespread amongst Gram-negative bacteria. *K. pneumoniae* possesses cellulose biosynthesis genes, including *bcsA*. However, as a non-motile organism that lacks flagella, *K. pneumoniae* does not encode a YcgR homolog.

Mutations within the PilZ domain of the MrkH protein completely destroy its activity, resulting in the loss of ability for *K. pneumoniae* to produce type 3 fimbriae and biofilms [10], [11], [12]. Furthermore, using an electrophoretic mobility shift assay (EMSA), we have demonstrated that the binding of MrkH to the *mrkA* promoter *in vitro* requires the presence of c-di-GMP, indicating that c-di-GMP is an effector essential for MrkH function [10]. The MrkH-*mrkA* regulatory system appears to represent one of the most efficiently regulated transcriptional switches in bacteria, but the mode of action of MrkH at its cognate target promoter has not been characterized. In this study, we investigated the mechanism by which MrkH activates *mrkA* transcription by analyzing the interaction of MrkH with both its DNA target and RNA polymerase.

## Materials and Methods

### Bacterial strains, plasmids and growth conditions

The bacterial strains and plasmids used in this study are described in Table S1. *K. pneumoniae* strain AJ218 (capsule serotype K54) is a human, urinary tract infection isolate [33]. Unless otherwise stated, bacteria were maintained in Luria-Bertani (LB) medium overnight at 37°C with shaking. When appropriate, media were supplemented with antibiotics at the following concentrations: ampicillin, 100 mg/mL; kanamycin, 50 mg/mL; chloramphenicol, 30 mg/mL (for *E. coli*) and 80 mg/mL (for *K. pneumoniae*); and trimethoprim, 40 mg/mL.

### DNA manipulation techniques

PCR amplifications were performed using GoTaq Green Master Mix (Promega, Madison, WI), Phusion Flash High-Fidelity PCR Master Mix (Finnzymes, Finland) or Vent DNA Polymerase (New England Biolabs, Ipswich, MA). Restriction endonucleases and T4 DNA ligase were obtained from New England Biolabs. Synthetic oligonucleotides for PCR (Table S2) were obtained from GeneWorks (Australia).

### Site-directed mutagenesis

Mutation in the *mrkA* promoter region and the *mrkH* gene were constructed by overlapping-extension PCR [34] of wild-type *mrkA* and *mrkH* DNA template using mutagenic oligonucleotides (Table S2). Overlapping primers were used together with the relevant upstream or downstream complementation primer. Amplified fragments were cloned into the TOPO-TA vector and sequenced. The mutant *mrkA* fragments were each cloned from the TOPO-TA derivatives into plasmid pMU2385 [35] to create *mrkA* promoter-*lacZ* fusions. The mutant *mrkH* genes were each cloned from the TOPO-TA derivatives into the *tet* gene of pACYC184 [36].

### β-galactosidase assay

β-galactosidase activity was assayed as described elsewhere [37]. The overnight cultures of the *E. coli* MC4100 [38] transformants were diluted 1∶25 in LB medium containing appropriate antibiotics and IPTG (0.5 mM) and grown at 37°C to OD_600_ = 0.6, after which the β-galactosidase activities were assayed. The data shown are the results of three independent assays.

### Expression and purification of the wild type and mutant MrkH-8×His proteins

The coding regions of the wild type and various mutant *mrkH* gene flanked by *Nde*I and *Bam*HI sites were PCR amplified using primer pairs mrkH(NdeI)11a and mrkH(BamHI)11a. The amplified DNA fragments were cloned into TOPO-TA and sequenced. The *mrkH* fragments encoding the wild type and mutant MrkH proteins with eight histidine residues tagged at the C-terminal end were then excised and cloned into the *Nde*I and *Bam*HI sites of pET11a (Novagen, Madison, WI) to form pET11a-mrkH-8His. For over-expression of His-tagged proteins, *E. coli* expression strain BL21(DE3) [39] containing pET11a derivatives was induced with 0.3 mM isopropyl-β-D-thiogalactopyr-anoside (IPTG) for 3 h at 20°C. Over-expressed proteins were purified using Metal Affinity Chromatography.

### Electrophoretic mobility shift assay (EMSA)

Primer mrkA116 was labelled at the 5′ end with [γ-32P]ATP and T4 polynucleotide kinase. The DNA fragments containing the wild type and mutant *mrkA* regulatory region were generated by PCR using primers ^32^P-mrkA116 and mrkA-155, with TOPO-TA derivatives carrying the *mrkA* promoter fragments (wild-type and mutants) as template. ^32^P-labelled *mrkA* fragments was incubated with varying amounts of purified His-tagged MrkH proteins (wild-type and mutants) with 200 µM c-di-GMP at 30°C for 20 min in the binding buffer (10 mM Tris HCl [pH 7.4], 50 mM KCl, 1 mM DTT, 100 mg/mL BSA and 5 ng/mL poly[dI-dC]). Glycerol was added to a final concentration of 6.5%. Samples were analyzed by electrophoresis on 5% native polyacrylamide gels (37.5∶1) containing 50 µM c-di-GMP. Electrophoresis was carried out at room temperature for approximately 8 h at 10 V/cm.

### qrt-RT-PCR

The various *K. pneumoniae* derivatives were grown in LB until OD_600_ = 0.7. Ten milliliters of culture was incubated with 20 ml of RNAprotect solution (Qiagen) at room temperature for 15 min. Cells were pelleted and RNA was purified using a FastRNA Pro Blue Kit (Q-BIOgene). The RNA samples were then treated with DNase I using a RNase-Free DNase Set (Qiagen) before being further purified using the RNeasy MiniElute Cleanup Kit (Qiagen). cDNA synthesis was carried out using Super-script II reverse transcriptase (Invitrogen), Random Primers (Invitrogen) and 10 µg of total RNA as specified by the supplier. Each 25 µl of qrt-RT-PCR reaction contained 10 ng cDNA, 300 nM of the specific primers (Table S2) and 12.5 µl 2× SYBR green master mix (Applied Biosystems). Data were normalized to the *K. pneumoniae* house-keeping gene *rpoD* and the relative expression ratio of a target gene was calculated using the method described by Pfaffl [40].

### Static biofilm assays

Biofilm assays were performed as described with minor modifications [10], [41]. Stationary-phase cells were sub-cultured 1∶100 in M63B1-GCAA minimal media (containing 1% glycerol and 0.3% casamino acids) in duplicate 96-well, flat bottom, non-tissue culture treated, polystyrene microtiter plates (Nunc, Penfield, NY). Following 24 h static incubation at 37°C, planktonic bacteria were decanted and wells were washed twice with distilled water. Biofilms were stained with 0.1% (wt/vol) crystal violet solution (Sigma-Aldrich), solubilized with 33% acetic acid and subsequently quantified by measuring the optical density at 595 nm. Data shown are the average values of two independent experiments.

### Hemagglutination assays

The presence of type 3 fimbriae was determined by mannose resistant hemagglutination (MRHA) assays, as detailed previously [10]. Tannic acid-treated human erythrocytes were mixed with equal volumes of a series of 2-fold dilutions of bacterial suspension with or without 4% D-mannose (Sigma-Aldrich). The minimum bacterial density (CFU/ml) required to agglutinate erythrocytes was measured.

### Western blot

MrkH-8×His expression (from pACYC184 derivatives) was detected by Western blot analysis using α-His antibody (Dianova, Germany) at a concentration of 1∶400. Whole cell lysates were prepared from overnight cultures. Samples were separated by sodium dodecyl sulphate (SDS)-polyacrylamide gel electrophoresis (PAGE) and transferred to Hybond-C Extra nitrocellulose (Amersham Biosciences, Sweden) using a Trans-Blot SD Electrophoretic Transfer Cell (Bio-Rad Laboratories, Hercules, CA) at 12 V for 30 min. Anti-mouse HRP (Biorad) was used as the secondary antibody at a concentration of 1∶8,000. Membranes were developed with ECL Western Blotting Detection Reagents (Amersham Biosciences) or TMB Membrane Peroxidase Substrate (KPL, Gaithersburg, MD).

## Results

### Identification of the MrkH binding site in the *mrkA* regulatory region

**Figure 1:**
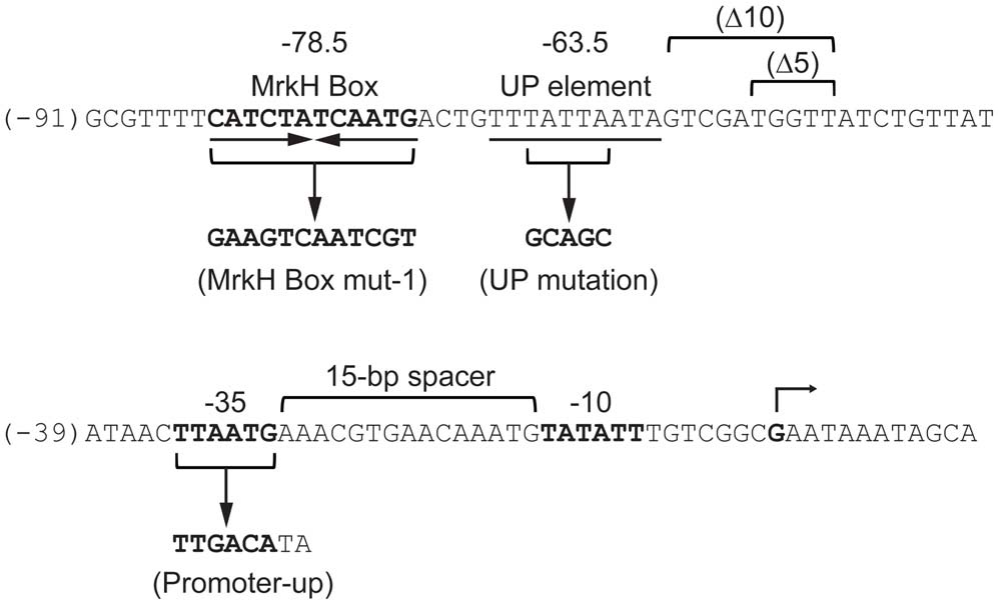
Nucleotide sequence of the *mrkA* regulatory region. The transcriptional start site of the *mrkA* promoter [10] is marked with an angled arrow. The numbering on the left of the sequence (in brackets) is relative to the start site of transcription. The −35 and −10 sequences are shown in bold. The spacer, the MrkH box (bold) and the putative UP element (underlined) are marked. The positions of the centers of the MrkH box and the AT-rich cluster (relative to the start site of transcription) are shown above or below the sequence. The genetic changes of the various mutations are shown below or above the sequence.

Because of the high degree of conservation between the various components of the RNA polymerases of *K. pneumoniae* and *E. coli* (http://ecocyc.org), analysis of transcriptional regulation of *K. pneumoniae* genes can be conveniently carried out in *E. coli* [9], [10], [42]. Furthermore, sequence analysis of *E. coli* genomes confirmed the absence of any sequences that could encode MrkH homologous. Using the *E. coli* K12 strain MC4100, we sought to map the MrkH binding site, and generated four PCR fragments of the *mrkA* regulatory region: from positions −84, −77, −71 or −67 to +166, relative to the start site of transcription (Fig. 1 and Table 1). These four fragments were each ligated into the single copy plasmid pMU2385 [10] to create *mrkA–lacZ* transcriptional fusions (*mrkA*-*lacZ*-2–5). The resulting pMU2385 derivatives, along with the previously constructed *mrkA–lacZ*-1 fusion (-91 to +166), were each transformed into *E. coli* MC4100 containing either the control plasmid pACYC184 (MrkH^−^ background), or the plasmid pMrkH (MrkH^+^ background) [10]. β-galactosidase levels were assessed for each of the transformants grown in LB at 37°C.

**Table 1:** Identification of the MrkH box by mutagenesis.

	*mrkA* promoter activity (Miller units)^a^	
*mrkA*-*lacZ* fusion	MrkH^−^	MrkH^+^
control (pMU2385)	0.2	0.2
*mrkA*-*lacZ*-1 (−91 to +166)	10	3886 (388)^b^
*mrkA*-*lacZ*-2 (−84 to +166)	11	4035 (367)
*mrkA*-*lacZ*-3 (−77 to +166)	42	52 (1)
*mrkA*-*lacZ*-4 (−71 to +166)	39	42 (1)
*mrkA*-*lacZ*-5 (−67 to +166)	34	33 (1)
MrkH box mut-1	30	33 (1)

a β-galactosidase assays were carried out using *E. coli* MC4100 derivatives after growth in LB. β-galactosidase activity is the average of three independent experiments, with standard deviation below 15%.

b Shown in parentheses are the values of fold activation, equal to the specific activity of β-galactosidase of the MrkH^+^ strain divided by that of the MrkH^−^ strain.

As shown in Table 1, the construct *mrkA–lacZ*-1 (−91 to +166) expressed 10 U and 3886 U of β-galactosidase activities in the MrkH^−^ and MrkH^+^ backgrounds, respectively, which represents 388-fold activation of *mrkA* expression by the MrkH protein. The very low levels of β-galactosidase activity in the MrkH^−^ background suggests that there exists no endogenous *E. coli* proteins that can induce *mrkA* expression. While *mrkA–lacZ*-2 (−84 to +166) exhibited the same level of MrkH-mediated activation as that of *mrkA–lacZ*-1, the expression of three other deletion mutants (*mrkA–lacZ*-3, *mrkA–lacZ*-4 and *mrkA–lacZ*-5) were not activated by MrkH. These results indicate that the region immediately downstream from position −84 contains the *mrkA* operator. Sequence analysis of this region revealed a 12-bp palindromic sequence between positions −84 and −73 (Fig. 1). To determine whether this sequence, which we named the ‘MrkH box’, is important for MrkH-mediated activation of *mrkA* transcription, we made a *mrkA* regulatory region mutation in which the DNA sequence of the MrkH box was scrambled (Fig. 1). A β-galactosidase assay showed that the MrkH box mut-1 mutation caused a complete loss of *mrkA* transcription activation (Table 1).

**Figure 2:**
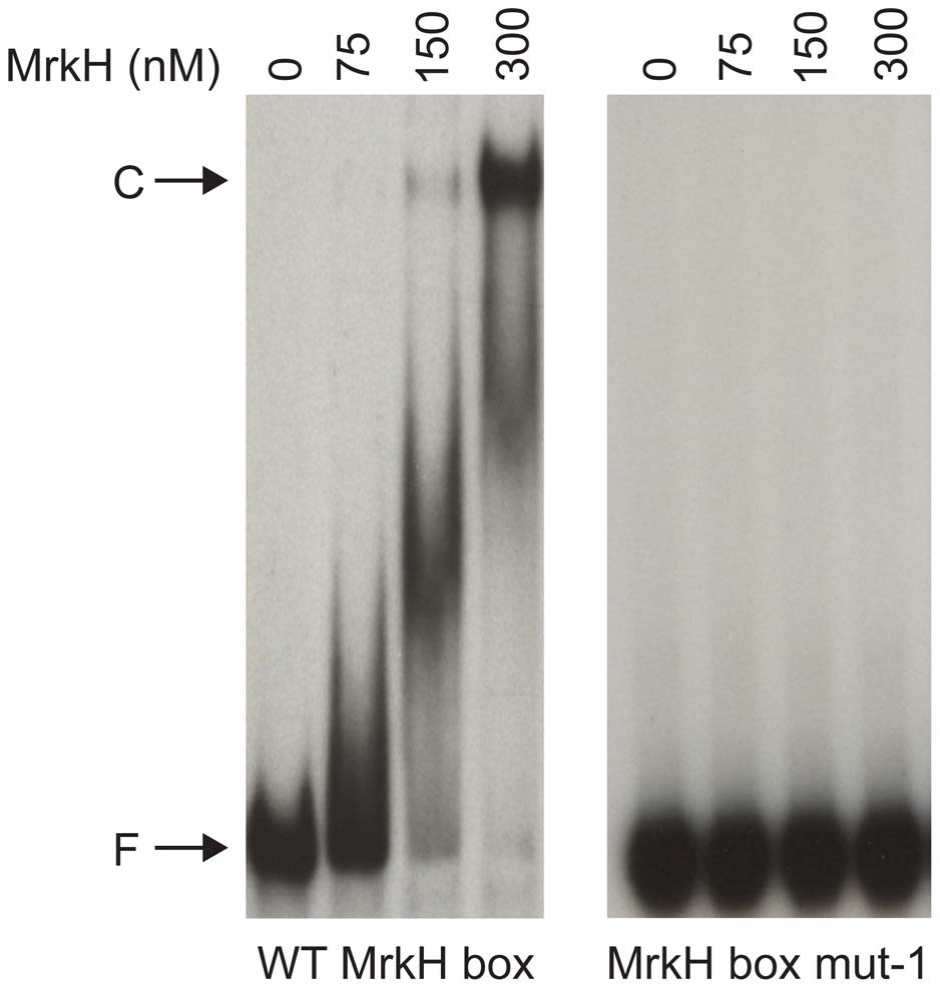
Analysis of MrkH-8×His binding to the wild-type MrkH and mutant *mrkA* fragments by EMSA. The two *mrkA* fragments (wild-type and MrkH box mut-1) spanning from −155 and +116 were each amplified and labeled at the 5′ end with ^32^P by PCR, using primer pairs ^32^P-mrkA116 and mrkA-155. DNA fragments were each mixed with varying amounts of MrkH-8×His in the presence of 50 µM c-di-GMP. Following incubation at 30°C for 20 min, samples were analyzed on native polyacrylamide gels. F: free DNA. C: protein-DNA complex.

To further confirm that the MrkH box is responsible for MrkH binding, we carried out an electrophoretic mobility shift assay (EMSA). Two ^32^P-labelled *mrkA*-promoter DNA fragments which contain the wild-type and the mutant MrkH boxes were each mixed with varying concentrations of the purified MrkH-8×His protein in the presence of c-di-GMP for 20 min at 30°C, after which the samples were analyzed on native polyacrylamide gels. The results in Fig. 2 show that the wild-type MrkH box DNA, but not the mutant *mrkA* fragment, is recognized by MrkH to form a protein-DNA complex, indicating that the MrkH box sequence is critical for MrkH to bind to the *mrkA* regulatory region.

**Figure 3:**
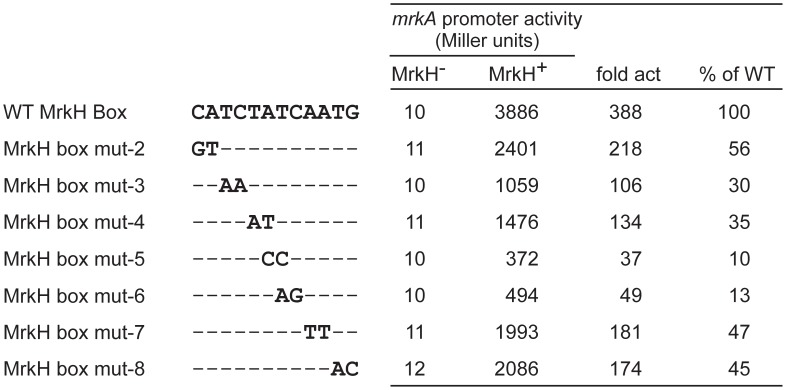
β-Galactosidase expression of mutant *mrkA-lacZ* fusions in the MrkH^-^ (MC4100 + pACYC184) and MrkH^+^ (MC4100 + pMrkH) backgrounds. The double-base substitutions in the various operator mutants are shown below the MrkH box sequence. The β-galactosidase activity shown is the mean of results from three independent experiments, with standard deviations below 15%. The fold activation (fold act) is the value for the β-galactosidase activity of the MrkH^+^ strain divided by that of the MrkH^−^ strain. The fold activation for each mutant is also expressed as the percentage of that of the wild-type (% of WT).

To evaluate the contribution of the nucleotides within the MrkH box to transcriptional activation of the *mrkA* promoter by MrkH, we made a series of double-base changes in the MrkH box (Fig. 3). β-galactosidase analysis showed that all of the operator mutations significantly affected MrkH-mediated activation. While five of these mutants (MrkH box mut-2, 3, 4, 7 and 8) exhibited a relatively small reduction in the levels of MrkH-mediated activation of *mrkA* expression (to 30–56% of the wild-type *mrkA*-*lacZ* level), the other two (MrkH box mut-5 and 6) which carry base changes in the center of the MrkH box had a stronger effect on activation by MrkH (10–13% of the wild-type *mrkA*-*lacZ* level) (Fig. 3). DNA fragments containing these two mutations (MrkH box mut-5 and 6) were analyzed by EMSA and the data in Fig. S1 confirmed that the mutant DNA fragments had a marked decrease in the affinity for the MrkH protein.

### Mutational analysis of the *mrkA* promoter

The *mrkA* promoter is composed of an imperfect −35 element (TTAATG) and a suboptimal spacer (15 bp) [10], which would explain the very weak *mrkA* promoter activity in a MrkH^−^ background (Fig. 1 and Table 2). To test whether the mechanism of MrkH action is to correct the *mrkA* promoter defects arising from its suboptimal core elements, we constructed a ‘promoter-up’ mutation, in which the −35 element was altered to the consensus sequence (TTGACA), and the spacer region increased to the optimum length of 17 bp (Fig. 1). The transcriptional activity of the mutant promoter (Promoter-up) was then compared with that of the wild-type *mrkA* promoter. In the MrkH^−^ background, the Promoter-up mutation caused a large increase in promoter activity, from 11 U to 3379 U (Table 2). In contrast, in the MrkH^+^ background, the mutant promoter had only a minor enhancement of transcriptional activity, from 4371 U to 5414 U (Table 2). The observation that the wild-type, but not the Promoter-up mutant *mrkA* promoter, required MrkH for maximal expression supports the hypothesis that MrkH functions to enhance the weak interaction between RNA polymerase and the wild-type *mrkA* promoter.

**Table 2:** The effects of various mutations on MrkH-dependent and -independent transcription of the *mrkA* promoter.

	*mrkA* promoter activity (Miller units)^a^	
*mrkA*-*lacZ* fusion	MrkH^−^	MrkH^+^
Wild type *mrkA* b	11	4371 (397)^c^
Promoter-up	3379	5414 (2)
Δ5	16	19 (1)
Δ10	17	2696 (159)
UP mutation	11	252 (23)

a β-galactosidase assays were carried out using *E. coli* MC4100 derivatives after growth in LB. β-galactosidase activity is the average of three independent experiments, with standard deviation below 15%.

b The pMU2385 derivative contains the WT *mrkA* regulatory region spanning the positions −190 and +166. All the other mutations described in this Table are based on this plasmid.

c Shown in parentheses are the values of fold activation, equal to the specific activity of β-galactosidase of the MrkH^+^ strain divided by that of the MrkH^−^ strain.

To investigate whether the MrkH-mediated activation of *mrkA* expression involves an interaction between MrkH and RNA polymerase (RNAP), we made two deletion mutations (Δ5 and Δ10) in the *mrkA* regulatory region (Fig. 1). Functional analysis of these mutations showed that, whereas deleting a full turn of DNA helix (10-bp; Δ10) between the MrkH Box and the *mrkA* promoter core elements had little effect on MrkH-mediated activation, removing half a turn of DNA helix (5-bp; Δ5) rendered the *mrkA* promoter insensitive to activation by MrkH. This “face-of-the-helix” specificity for maximal activation is consistent with the conclusion that an interaction between MrkH and RNAP occurs and that such interaction requires a proper alignment of the two proteins on the *mrkA* promoter region. Similar face-of-the-helix effects were also seen for the CRP-mediated activation of its target promoters [43], [44].

The *mrkA* upstream region contains an AT-rich cluster centered at −63.5, which could function as an UP element [45] for *mrkA* transcriptional initiation (Fig. 1). To determine if this AT-rich cluster plays any role in *mrkA* transcription, we replaced four AT pairs by GC pairs within this 10-bp sequence (UP mutation) (Fig. 1). While, in comparison with the wild-type promoter, this mutation exhibited no change in transcription levels in the MrkH^−^ background, the activity of the mutant promoter was decreased to 252 U in the MrkH^+^ background, representing a major reduction in the degree of *mrkA* transcription (from 397-fold to 23-fold). These results clearly showed that the AT-rich cluster centered at −63.5 is involved in MrkH-mediated activation of *mrkA* transcription. Furthermore, an EMSA experiment showed that, similar to the wild-type *mrkA* fragment, MrkH was able to completely shift the mutant *mrkA* fragment (UP mutation) at 300 nM (Fig. S2), indicating that this AT-rich cluster is not important for MrkH binding.

### qrt-RT-PCR analysis of promoter and operator mutations of *mrkA*

To confirm the effect of the *mrkA* promoter and operator mutations in *K. pneumoniae*, we carried out qrt-RT-PCR analysis. To do this, three pMU2385 derivatives containing different *mrkA* fragments (wild-type, Promoter-up and MrkH box mut-1) were each introduced into a Δ*mrkH* mutant *K. pneumoniae* strain carrying either pACYC184 (MrkH^−^) or pMrkH (MrkH^+^). Following growth of the various *K. pneumoniae* strains to mid-log phase, total cellular RNA was isolated from each of these strains and the *mrkA* transcripts encoded by the pMU2385 derivatives was probed by using primers mrkA15F and pMU2385Rev. The levels of expression from each sample were normalized to the reference gene *rpoD* and the relative abundance of transcripts from the MrkH^−^ and MrkH^+^ backgrounds was assessed. As shown in Table 3, the transcription of the wild-type *mrkA* promoter was up-regulated 637-fold in the MrkH^+^ background compared with the value in the MrkH^−^ background. In contrast, less than 2-fold activation by MrkH was detected for the *mrkA* promoter mutant (Promoter-up) and no significant activation by MrkH was seen for the *mrkA* operator mutant (MrkH box mut-1). These results are in agreement with those obtained by β-galactosidase assays (see above).

**Table 3:** Relative expression of the wild-type and mutant *mrkA* promoters in MrkH^+^ and MrkH^−^ backgrounds of *K. pneumoniae*.

K. pneumoniae (MrkH^+^/MrkH^−^)	Relative expression ratio^a^
Wild-type mrkA promoter	636.93±47
MrkH box mut-1	1.15±0.06
Promoter-up mutant	1.86±0.07

a Mean expression of transcripts from the different *mrkA-lacZ* fusions in the MrkH^+^ background relative to the MrkH^−^ background. The values are the mean ± SEM of three biological replicates.

### RNAP and positive control of the *mrkA* promoter

**Figure 4:**
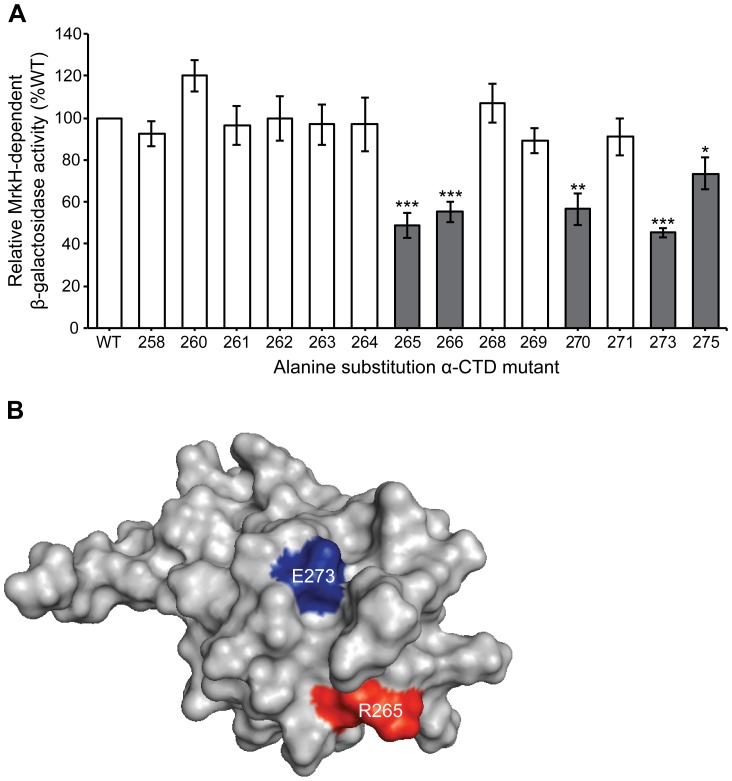
Identification of amino acid residues between positions 258 and 275 of the RNAP α-CTD important for MrkH-mediated activation of *mrkA* transcription *in vivo*. (A) A set of plasmids carrying *rpoA* mutations with alanine replacement between positions 258 and 275 were each transformed into *E. coli* strain MC4100 containing plasmids *mrkA-lacZ*(−190 to +166) and pMrkH. The β-galactosidase activities of the various samples (from three independent experiments) are presented relative to the activity of the strain carrying the plasmid pLAW2 containing wild-type *rpoA*. Grey bars correspond to those alanine replacements that result in a reduction of at least 20% of β-galactosidase activity. Error bars indicate standard deviation of the mean. Data are representative of three independent experiments. Statistical significance between wild-type and alanine mutants was analysed by Student's *t*-test, where ***  =  *P*<0.001, **  =  *P*<0.01, *  =  *P*<0.05. (B) The structure of α-CTD of RNAP [52], [59] is shown and the surface-exposed residues R265 (red) and E273 (blue) are marked.

Three observations collectively implicate a role for the C-terminal domain of the α subunit (α-CTD) of RNA polymerase in the positive control of the *mrkA* promoter: (i) the upstream location of the MrkH box, (ii) the “face-of-the-helix” specificity of MrkH-mediated activation, and (iii) the presence of a putative UP element in the *mrkA* promoter. To test this hypothesis, we investigated whether overexpression of *rpoA* mutations affected MrkH-mediated activation of *mrkA* expression. Plasmid pLAW2 [46] which carries the wild-type *E. coli rpoA* gene (under the control of the *lpp/lacUV5* promoter) and a set of pLAW2 derivatives, in which the *rpoA* gene contains alanine substitutions at positions 258 to 275, were each transformed into *E. coli* strain MC4100 containing pMrkH and the wild-type *mrkA-lacZ* fusion pMU2385 derivative (*mrkA* promoter positions −190 to +166). β-galactosidase assays showed that overexpression of the α subunit variants R265A, S266A, L270A, E273A and I275A led to a significant reduction of the MrkH-dependent *mrkA* expression (Fig. 4A). Moreover, Western blot analysis showed that MrkH was efficiently expressed in the MC4100 backgrounds over-expressing either the wild-type or each of the five mutant α subunits (Fig. S3). These results suggest that the residues at these positions of the wild-type α-CTD are involved in the MrkH-mediated transcriptional activation of the *mrkA* promoter.

### A positive control mutation in MrkH

**Figure 5:**
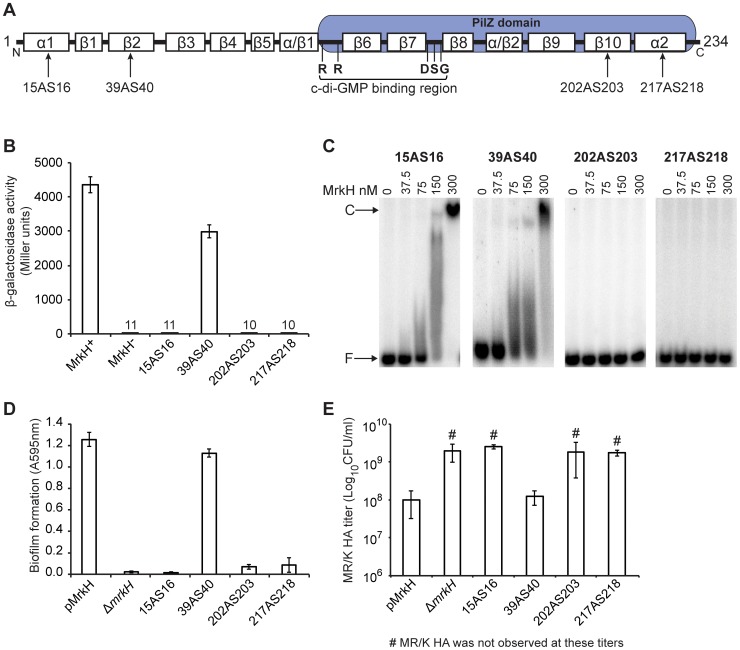
Analysis of MrkH regions required for transcriptional activation and DNA binding. (**A**) Predicted secondary structure of MrkH α-helices and β-strands using programs SOPMA, JPred, PSIPRED and PROF [60], [61], [62], [63]. Consensus could not be identified for two regions (α/β1 and α/β2). The positions and amino acid (AS) insertions of four MrkH mutants constructed are shown. The c-di-GMP binding region containing the RxxxR and DxSxxG motifs is shown. (**B**) β-galactosidase assays performed with *E. coli* MC4100 containing the plasmid *mrkA-lacZ*(−190 to +166) (MrkH^−^) and either wild-type pMrkH (MrkH^+^) or mutant pMrkH constructs containing AS insertion mutations in the indicated positions. Values represent the mean of three replicate samples. The error bars represent the standard deviation. Statistical significance was identified between wild-type and all mutant MrkH constructs (Student's *t*-test; *P*<0.001). (**C**) EMSA analysis of mutant forms of purified MrkH-8×His (MrkH: 15AS16, 39AS40, 202AS203 and 217AS218) binding to the wild-type MrkH box. F: free DNA. C: protein-DNA complex. (**D**) Biofilm formation assays performed with *K. pneumoniae* AJ218 Δ*mrkH* mutant strains (containing wild-type pMrkH, or mutant pMrkH constructs containing AS insertion mutations). Values represent eight replicate sample wells for each strain performed in two independent experiments. The error bars represent the standard deviation. Statistical significance was identified between wild-type and all mutant MrkH constructs (Student's *t*-test; *P*<0.001). (E) MR/K HA assay of the indicated *K. pneumoniae* strains (explained above). Values represent the mean of three independent experiments.

Although the central region of MrkH is known to contain conserved residues within the PilZ domain that are responsible for c-di-GMP binding and MrkH function [10], [11], [12] (Fig. 5A), the transcriptional activation and DNA-binding domains of MrkH have not yet been identified. Analysis of the secondary structure of MrkH using several algorithms revealed the presence of an α-helix and several β-sheets at both the N-terminus and C-terminus of the protein. To characterize these two regions, we generated four mutations where alanine and serine (AS) residues were inserted into α1, β2, β10 and α2 (Fig. 5A). Insertion mutagenesis of AS was used to characterize the α subunit of the *E. coli* RNA polymerase and the RegA regulator of *C. rodentium* as insertion of these amino acids does not cause major perturbation in the overall structure of proteins [47], [48].

*In vivo* transcriptional analysis showed that disruption of β2 (39AS40) had little effect on MrkH-mediated activation of *mrkA* expression (Fig. 5B), indicating that the β2 region of MrkH is not involved in DNA-binding or the possible interaction with RNA polymerase. In contrast, the amino acid insertions into α1, β10 and α2 (15AS16, 202AS203 and 217AS218) rendered the MrkH protein completely defective in transcriptional activation (Fig. 5B). Western blot analysis showed the mutant proteins to be stably expressed (Fig. S4). To further characterize the four mutations, they were each purified as C-terminal 8×His-tag proteins. EMSA analysis using a ^32^P-labelled wild-type *mrkA* fragment and each of the mutant MrkH proteins showed that, whereas 15AS16 and 39AS40 retained DNA-binding activity, 202AS203 and 217AS218 were unable to interact with the DNA target (Fig. 5C). This suggests that the N-terminal tip of the MrkH protein is responsible for the positive control of the *mrkA* promoter and the C-terminal region is either directly or indirectly involved in DNA-binding. The ability of the four mutant MrkH constructs to mediate biofilm formation via type 3 fimbriae expression in *K. pneumoniae* was also assessed. In agreement with the data from the transcriptional analysis as shown in Fig. 5B, mutation of the α1, β10 and α2 regions resulted in the loss of biofilm formation and type 3 fimbriae expression, while disruption of β2 caused little change in the ability of MrkH to promote high levels of biofilm formation and type 3 fimbriae expression (Fig. 5D & E).

## Discussion

The data obtained from the present study revealed the mechanism by which the MrkH protein controls the expression of the *mrkA* promoter, a key point of regulation that is critical for the switch between planktonic growth and biofilm formation of *K. pneumoniae*. The *mrkA* promoter is essentially transcriptionally inactive in the MrkH^−^ background (Table 2) or in a *K. pneumoniae* strain that is defective in c-di-GMP synthesis [10], [11], [12]. The extremely low level basal activity of the promoter is attributed to the presence of a suboptimal −35 region (the sequence TTAATG versus the consensus TTGACA) and a shorter spacer (15-bp versus 17-bp) within the promoter core sequence. The −35 and the −10 hexamers are known to directly interact with amino acid residues within regions 4.2 and 2.4, respectively, of the σ^70^ subunit of RNAP [49], [50], therefore the base composition and a proper alignment of the two elements are critical for RNAP binding. Consistent with this model, inserting a consensus sequence in the −35 hexamer and increasing the length of the spacer (Promoter-up mutation) led to greater than 300-fold enhancement of the transcriptional activity of *mrkA* (Table 2).

Two *cis*-acting elements, a 12-bp palindromic sequence (the MrkH box) and a 10-bp AT-rich cluster (UP element) centered at −78.5 and −63.5, respectively, are important for MrkH-mediated activation of *mrkA* expression. Results from EMSA experiments indicate that the MrkH box is responsible for MrkH binding. Palindromic DNA sequences attract DNA-binding proteins in a dimer formation [51], suggesting that MrkH binds to its DNA target as a dimer. However, further biophysical analysis of the MrkH protein is required to test this model.

The “face-of-the-helix” effect between the MrkH box and the *mrkA* promoter core sequence demonstrated by deletion mutagenesis suggests a direct interaction between MrkH and RNAP. Using a set of *E.coli rpoA* variants that carry alanine replacements in the α-CTD of RNAP (the α-CTD of *E. coli* is identical to that of *K. pneumoniae*, http://ecocyc.org), we identified 5 residues (R265, S266, L270, E273 and I275) that are required for MrkH-mediated activation of *mrkA* expression. Based on the crystal structure of the α-CTD of RNAP [52], R265 and E273 are exposed on the surface of the α-CTD (Fig. 4B), while the other three residues are buried inside the structure and mutations in these three positions may impact indirectly on residues contacting MrkH. Conversely, the surface-exposed residues R265 and E273 are prime candidates to be directly involved in making contact with DNA and/or with the MrkH protein.

R265 has been shown to directly interact with the UP elements of many bacterial promoters [53], [54]. An elegant NMR study by Ishihama and Kyogoku groups showed that the guanidino group of R265 of the α-CTD interacts with the negatively charged phosphate backbone within the DNA minor groove of an UP element [55]. If R265 also contacts the UP element of the *mrkA* promoter, this interaction appears to contribute significantly to the MrkH-dependent transcription initiation of the *mrkA* promoter, as replacing AT pairs by GC pairs within the UP element led to a 17-fold reduction of the promoter activity in the MrkH^+^ background (Table 2). In the context of RNAP binding to the *proP* P2 promoter in *E. coli*, E273 has been shown to interact with the regulatory protein Fis [56]. Mutational analysis of the *mrkH* gene showed that its short N-terminal arm is involved in transcriptional activation of the *mrkA* promoter. However, detailed genetic, biochemical and structural studies are required to establish the mechanism of interaction between MrkH and RNAP.

**Figure 6:**
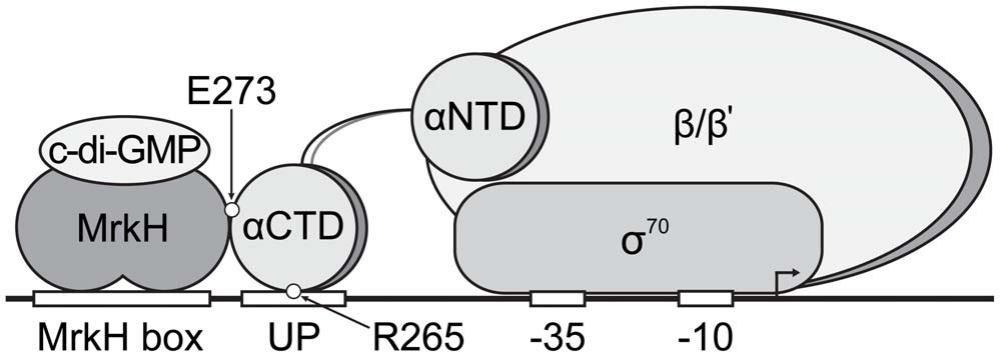
A putative model for MrkH-mediated activation of *mrkA* transcription. The cartoon depicts a three-way interaction between MrkH, the α-CTD of RNAP and the *mrkA* regulatory region, required for stimulation of the rate of transcription initiation. Upon binding effector c-di-GMP, the MrkH protein is able to bind to the MrkH box, triggering an interaction with the α-CTD of RNAP (via the E273 determinant). This interaction facilitates the binding of α-CTD (via the R265 determinant) to the *mrkA* UP element.

Based on the results of this study, we propose that MrkH functions as a Class I transcriptional activator at the *mrkA* promoter [57], [58]. Upon binding the MrkH box (in the presence of c-di-GMP) and through an interaction with the α-CTD, MrkH enhances the binding of RNAP to the UP and the core elements of the *mrkA* promoter, thereby stimulating the rate of transcription initiation (Fig. 6). Using this regulatory mechanism, *K. pneumoniae* is able to rapidly drive the production of type 3 fimbriae under desirable environmental conditions, leading to the rapid formation of biofilms.

## Supporting Information

Figure S1 EMSA analysis of the binding of purified MrkH-8×His to the *mrkA* fragment mutated in the MrkH box. ^32^P-labelled DNA fragments (WT MrkH box, MrkH box mut-5 and MrkH box mut-6) were each mixed with varying amounts of MrkH in the presence of c-di-GMP (200 µM) and following incubation at 30°C for 20 min, samples were analyzed on native polyacrylamide gels. F: free DNA. C: protein-DNA complex.
(TIF)

Figure S2 EMSA analysis of the binding of purified MrkH-8×His to the *mrkA* fragment mutated in the UP element. See the legend to Fig. S1 for experimental details. F: free DNA. C: protein-DNA complex.
(TIF)

Figure S3 Western blot analysis of MrkH expression in *E. coli* strain MC4100. MrkH was expressed as a C-terminal MrkH-8×His fusion from the plasmid pACYC184-*mrkH*-8×His in MC4100 which also carried a pLAW2 derivative expressing the wild-type or each of the mutant α subunits of RNAP (R265A, S266A, L270A, E273A and I275A). The MC4100 derivate carrying pACYC814 and pLAW2(WT *rpoA*) was used as the negative control. The induction of the different *rpoA* alleles was as described in the legend to Fig. 4. Western blot of MrkH-8×His was performed using α-His antibody.
(TIF)

Figure S4 Western blot analysis of MrkH expression in *E. coil* strain MC4100. Wild-type and mutant forms of MrkH-8×His were expressed from pACYC184 in MC4100. Samples were prepared by sonication followed by centrifugation and supernatants were separated by SDS-PAGE. Following transfer, the membrane was probed with α-His antibody. *E. coli* MC4100 harboring empty pACYC184 was used as the negative control.
(TIF)

Table S1 Bacterial strains and plasmids used in this study.
(DOCX)

Table S2 Oligonucleotide primers used in this study#. # Restriction endonuclease recognition sites are underlined. F/for, forward (5′) primer. R/Rev, reverse (3′) primer.
(DOCX)
