# Supplementary figures and images for: Transcriptional Activation of the mrkA Promoter of the Klebsiella pneumoniae Type 3 Fimbrial Operon by the c-di-GMP-Dependent MrkH Protein
Source: PLoS One. 2013 Nov 14;8(11):e79038. doi: 10.1371/journal.pone.0079038 (PMC3828302; doi:10.1371/journal.pone.0079038)

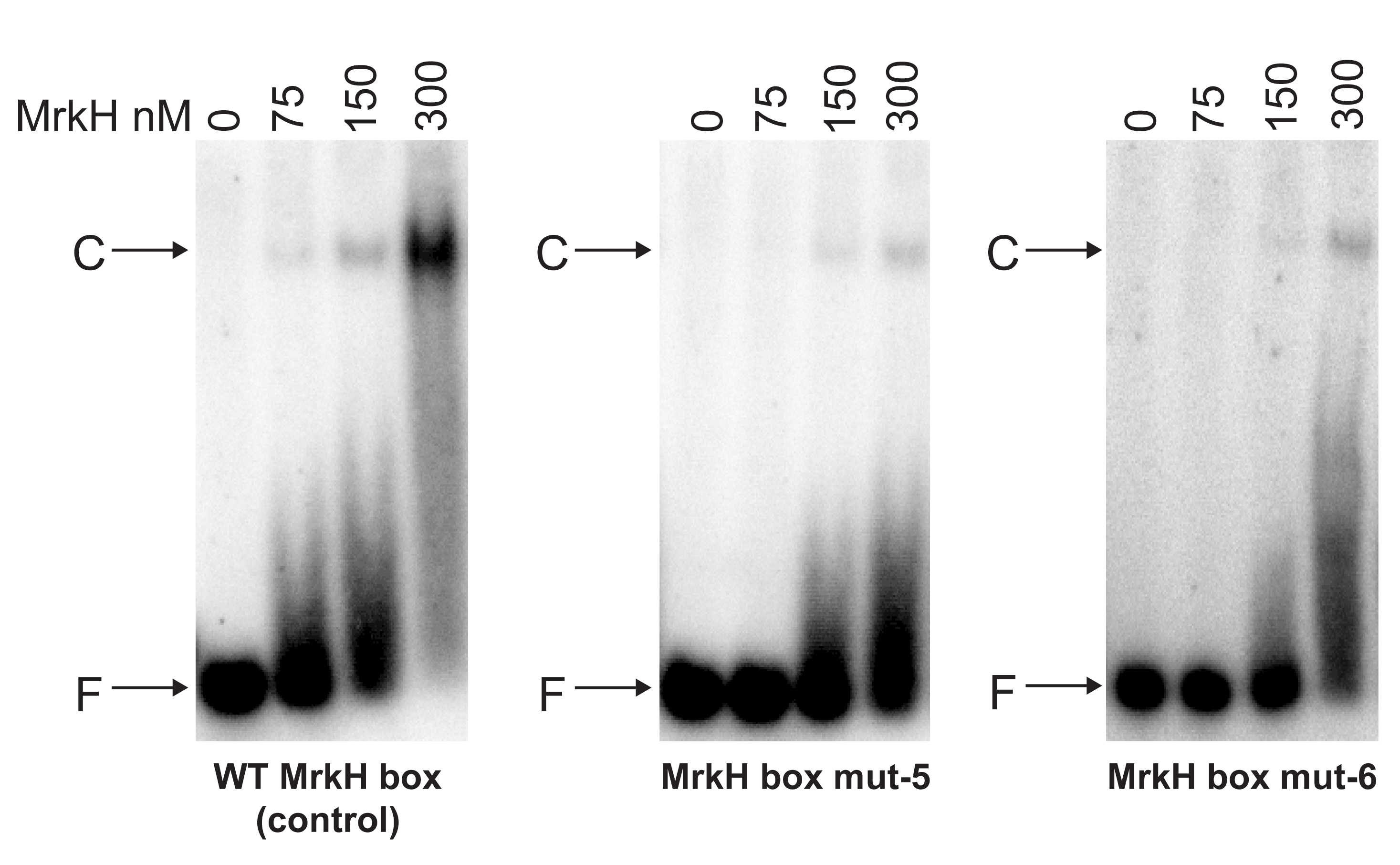

Supplement: Figure S1 — EMSA analysis of the binding of purified MrkH-8×His to the mrkA fragment mutated in the MrkH box. 32P-labelled DNA fragments (WT MrkH box, MrkH box mut-5 and MrkH box mut-6) were each mixed with varying amounts of MrkH in the presence of c-di-GMP (200 µM) and following incubation at 30°C for 20 min, samples were analyzed on native polyacrylamide gels. F: free DNA. C: protein-DNA complex. (TIF) [file pone.0079038.s001.tif]

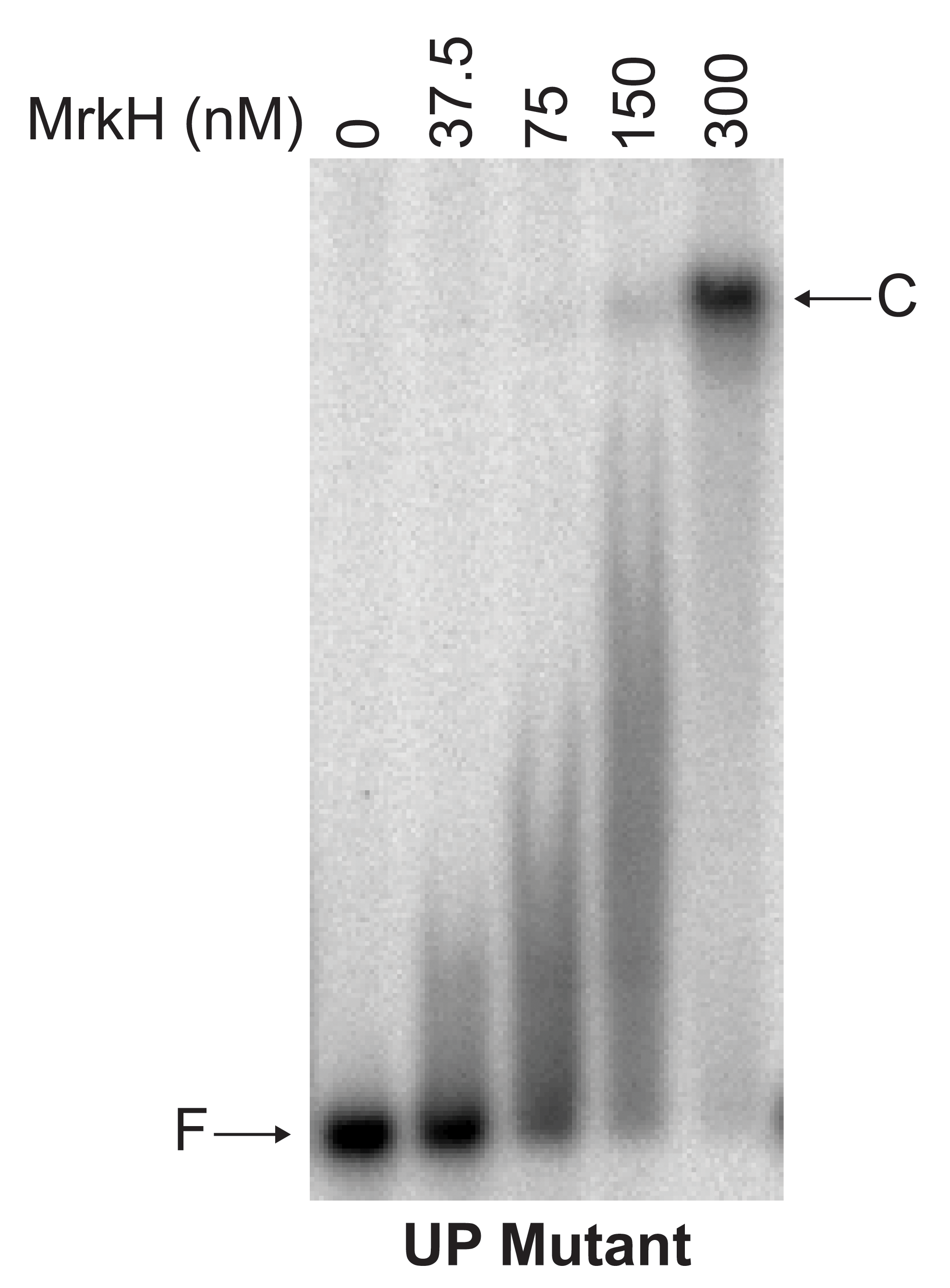

Supplement: Figure S2 — EMSA analysis of the binding of purified MrkH-8×His to the mrkA fragment mutated in the UP element. See the legend to Fig. S1 for experimental details. F: free DNA. C: protein-DNA complex. (TIF) [file pone.0079038.s002.tif]

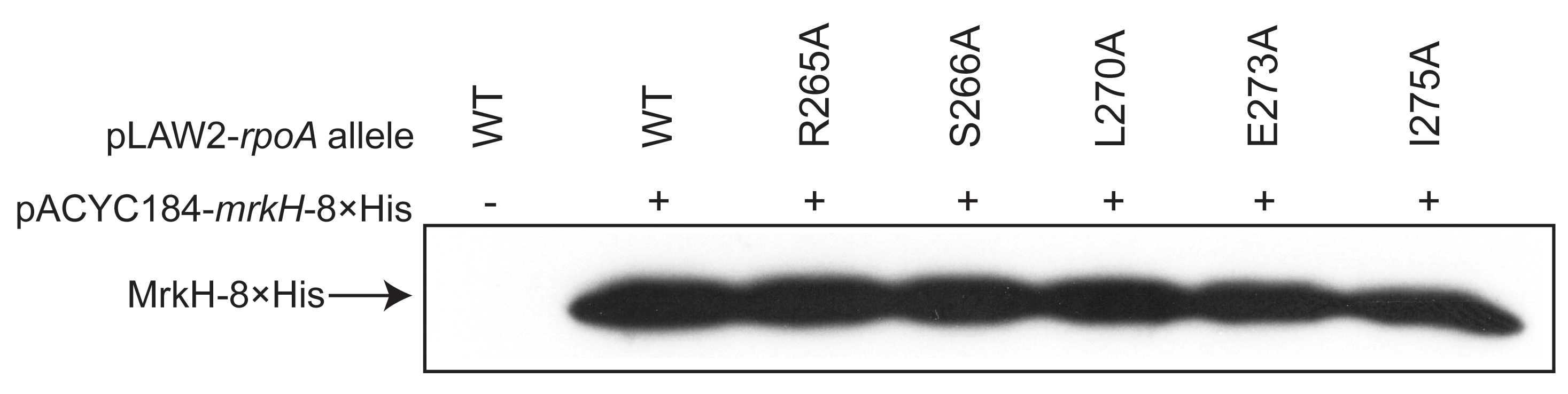

Supplement: Figure S3 — Western blot analysis of MrkH expression in E. coli strain MC4100. MrkH was expressed as a C-terminal MrkH-8×His fusion from the plasmid pACYC184-mrkH-8×His in MC4100 which also carried a pLAW2 derivative expressing the wild-type or each of the mutant α subunits of RNAP (R265A, S266A, L270A, E273A and I275A). The MC4100 derivate carrying pACYC814 and pLAW2(WT rpoA) was used as the negative control. The induction of the different rpoA alleles was as described in the legend to Fig. 4. Western blot of MrkH-8×His was performed using α-His antibody. (TIF) [file pone.0079038.s003.tif]

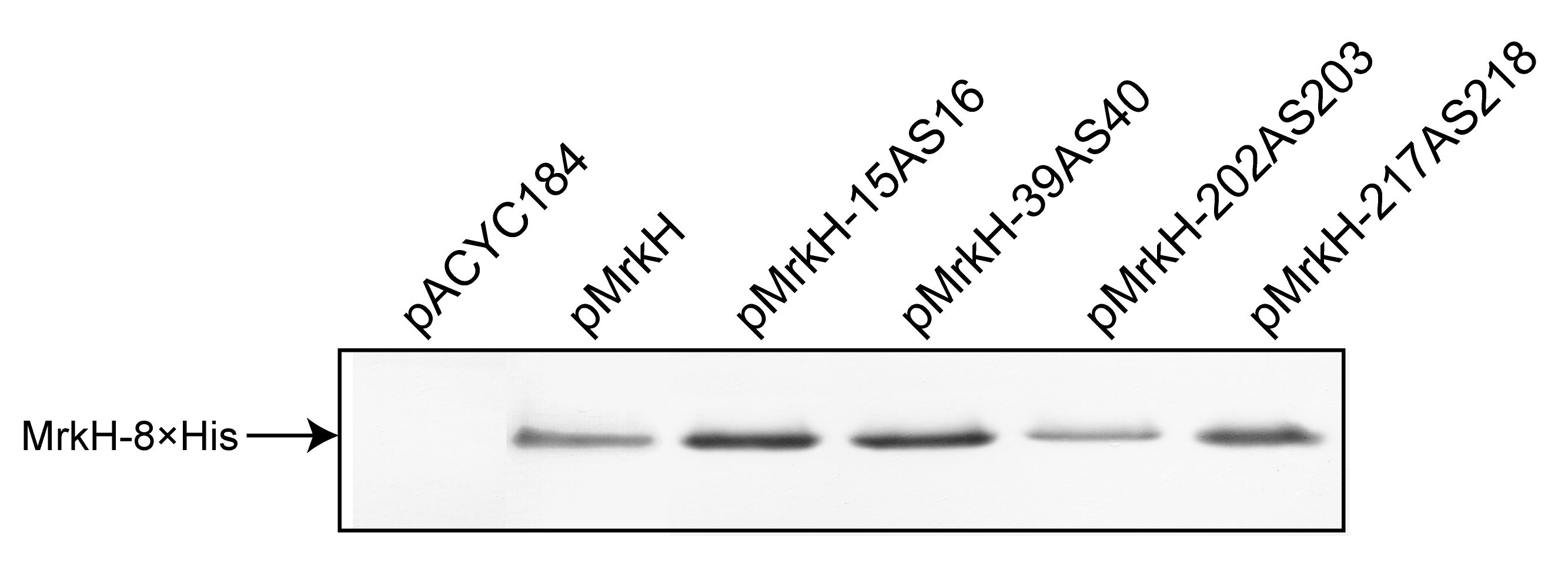

Supplement: Figure S4 — Western blot analysis of MrkH expression in E. coil strain MC4100. Wild-type and mutant forms of MrkH-8×His were expressed from pACYC184 in MC4100. Samples were prepared by sonication followed by centrifugation and supernatants were separated by SDS-PAGE. Following transfer, the membrane was probed with α-His antibody. E. coli MC4100 harboring empty pACYC184 was used as the negative control. (TIF) [file pone.0079038.s004.tif]
