# Supplementary material for: Transcriptional Activation of the mrkA Promoter of the Klebsiella pneumoniae Type 3 Fimbrial Operon by the c-di-GMP-Dependent MrkH Protein
Source: PLoS One. 2013 Nov 14;8(11):e79038. doi: 10.1371/journal.pone.0079038 (PMC3828302; doi:10.1371/journal.pone.0079038)
Supplement: Table S1 — Bacterial strains and plasmids used in this study. (DOCX) [file pone.0079038.s005.docx]

| **Strain or plasmid** | **Relevant phenotypes and genotypes** | **Source or reference** |
| --- | --- | --- |
| **Strains** |  |  |
| *K. pneumoniae* AJ218 | Wild-type, clinical isolate, serotype K54; Ap^R^ | [33] |
| *K. pneumoniae* Δ*mrkH* | AJ218 *mrkH* deletion mutant; Ap^R^ | [[10](#_ENREF_10)] |
| *E. coli* BL21(DE3) | F-, *omp*T, *hsd*S_B_(r_B_-, m_B_-), *dcm*, *gal*, λ(DE3) | [39] |
| MC4100 | Δ(*argF*-*lac*)*U169*, *rpsL150*, *relA*, *araD139*, *fib5301*, *deoC1*, *ptsF25* | [38] |
| **Plasmids** |  |  |
| TOPO-TA | High-copy-no. cloning vector for PCR products; Ap^R^ Km^R^ | Invitrogen |
| pMU2385 | *galK’*-*lac’Z*, IncW, single-copy-no. transcriptional-fusion vector; Tp^R^ | [35] |
| pACYC184 | Medium-copy-no. cloning vector, p15A ori; Tet^R^ Chl^R^ | [36] |
| pET11a | Expression vector using T7 promoter; Ap^R^ | Novagen |
| pLAW2 (and derivatives) | Plasmid carrying *rpoA* encoding RNAP α subunit (and derivatives carrying alanine substitutions at positions 258–275) | [49] |
| pET11a-*mrkH*-8×His | AJ218 *mrkH* tagged with 8×His at C-terminus cloned into pET11a; Ap^R^ | [[10](#_ENREF_10)] |
| pACYC184-*mrkH*-8×His | AJ218 *mrkH* tagged with 8×His at C-terminus cloned into pACYC184; Chl^R^ | This study |
| pMrkH | AJ218 *mrkH* cloned into pACYC184; Chl^R^ | [[10](#_ENREF_10)] |
| *mrkA-lacZ*(-190 to +166) | *mrkA-lacZ* transcriptional fusion vector (pMU2385) from *mrkA* promoter nucleotides -190 to +166 relative to the *mrkA* transcription start site; Tp^R^ | This study |
| *mrkA-lacZ*-1 | *mrkA-lacZ* transcriptional fusion vector (pMU2385) from *mrkA* promoter nucleotides -91 to +166 relative to the *mrkA* transcription start site; Tp^R^ | [[10](#_ENREF_10)] |
| *mrkA-lacZ*-2 | *mrkA-lacZ* transcriptional fusion vector (pMU2385) from *mrkA* promoter nucleotides -84 to +166 relative to the *mrkA* transcription start site; Tp^R^ | This study |
| *mrkA-lacZ*-3 | *mrkA-lacZ* transcriptional fusion vector (pMU2385) from *mrkA* promoter nucleotides -77 to +166 relative to the *mrkA* transcription start site; Tp^R^ | This study |
| *mrkA-lacZ*-4 | *mrkA-lacZ* transcriptional fusion vector (pMU2385) from *mrkA* promoter nucleotides -71 to +166 relative to the *mrkA* transcription start site; Tp^R^ | This study |
| *mrkA-lacZ*-5 | *mrkA-lacZ* transcriptional fusion vector (pMU2385) from *mrkA* promoter nucleotides -67 to +166 relative to the *mrkA* transcription start site; Tp^R^ | This study |
| MrkH box mut-1 | Derivative of *mrkA-lacZ*(-190 to +166) where the 12-bp MrkH box is mutated; Tp^R^ | This study |
| MrkH box mut-2 | Derivative of *mrkA-lacZ*(-190 to +166) where bases 1 and 2 (CA) of the MrkH box are replaced with GT); Tp^R^ | This study |
| MrkH box mut-3 | Derivative of *mrkA-lacZ*(-190 to +166) where bases 3 and 4 (TC) of the MrkH box are replaced with AA); Tp^R^ | This study |
| MrkH box mut-4 | Derivative of *mrkA-lacZ*(-190 to +166) where bases 5 and 6 (TA) of the MrkH box are replaced with AT; Tp^R^ | This study |
| MrkH box mut-5 | Derivative of *mrkA-lacZ*(-190 to +166) where bases 6 and 7 (AT) of the MrkH box are replaced with CC; Tp^R^ | This study |
| MrkH box mut-6 | Derivative of *mrkA-lacZ*(-190 to +166) where bases 7 and 8 (TC) of the MrkH box are replaced with AG; Tp^R^ | This study |
| MrkH box mut-7 | Derivative of *mrkA-lacZ*(-190 to +166) where bases 9 and 10 (AA) of the MrkH box are replaced with TT; Tp^R^ | This study |
| MrkH box mut-8 | Derivative of *mrkA-lacZ*(-190 to +166) where bases 11 and 12 (TG) of the MrkH box are replaced with AC; Tp^R^ | This study |
| Promoter-up | Derivative of *mrkA-lacZ*(-190 to +166) where both the -35 element and spacer region are replaced with a consensus sequence; Tp^R^ | This study |
| Δ5 | Derivative of *mrkA-lacZ*(-190 to +166) where 5 bp are deleted between the MrkH box and *mrkA* promoter core elements; Tp^R^ | This study |
| Δ10 | Derivative of *mrkA-lacZ*(-190 to +166) where 10 bp are deleted between the MrkH box and *mrkA* promoter core elements; Tp^R^ | This study |
| UP mutation | Derivative of *mrkA-lacZ*(-190 to +166) where the UP element at -64.5 is mutated; Tp^R^ | This study |
| 15AS16 | Derivative of pMrkH where AS-coding sequences are inserted between MrkH positions 15-16; Chl^R^ | This study |
| 39AS40 | Derivative of pMrkH where AS-coding sequences are inserted between MrkH positions 39-40; Chl^R^ | This study |
| 202AS203 | Derivative of pMrkH where AS-coding sequences are inserted between MrkH positions 202-203; Chl^R^ | Thus study |
| 217AS218 | Derivative of pMrkH where AS-coding sequences are inserted between MrkH positions 217-218; Chl^R^ | This study |
