# Supplementary material for: Transcriptional Activation of the mrkA Promoter of the Klebsiella pneumoniae Type 3 Fimbrial Operon by the c-di-GMP-Dependent MrkH Protein
Source: PLoS One. 2013 Nov 14;8(11):e79038. doi: 10.1371/journal.pone.0079038 (PMC3828302; doi:10.1371/journal.pone.0079038)
Supplement: Table S2 — Oligonucleotide primers used in this study#. # Restriction endonuclease recognition sites are underlined. F/for, forward (5′) primer. R/Rev, reverse (3′) primer. (DOCX) [file pone.0079038.s006.docx]

| **Primer name** | **Sequence (5’-3’)** |
| --- | --- |
| mrkA116 | GCCACTGCTAATAGAATCTTCGACTG |
| mrkA-155 | GCTGTTGCGGTCACTTTCTCTCGC |
| Upstream flanking (BamHI) | AGGATCCGCTGTTGCGGTCACTTTCTCTCGC |
| Downstream flanking (HindIII) | GAAGCTTCACACTGTGAATTCGCATAGAACCAG |
| Promoter-up (for) | CGATGGTTATCTGTTATATAACTTGACATAAACGTGAACAAATGTATATTTGTC |
| Promoter-up (rev) | GACAAATATACATTTGTTCACGTTTATGTCAAGTTATATAACAGATAACCATCG |
| Δ5 (for) | CATCTATCAATGACTGTTTATTAATAGGGTTATCTGTTATATAACTTAATGAAACG |
| Δ5 (rev) | CGTTTCATTAAGTTATATAACAGATAACCCTATTAATAAACAGTCATTGATAGATG |
| Δ10 (for) | CATCTATCAATGACTGTTTATTAATAATCTGTTATATAACTTAATGAAACG |
| Δ10 (rev) | CGTTTCATTAAGTTATATAACAGATTATTAATAAACAGTCATTGATAGATG |
| MrkH Box mut-1 (for) | CCACCCTCGCGTTTTGAAGTCAATCGTACTGTTTATTAATAGTCGATG |
| MrkH Box mut-1 (rev) | CATCGACTATTAATAAACAGTACGATTGACTTCAAAACGCGAGGGTGG |
| MrkH Box mut-2 (for) | TGGATCCGCGTTTTGTTCTATCAATGACTGTTTATTAATAGTCG |
| MrkH Box mut-3 (for) | TGGATCCGCGTTTTCAAATATCAATGACTGTTTATTAATAGTCG |
| MrkH Box mut-4 (for) | TGGATCCGCGTTTTCATCATTCAATGACTGTTTATTAATAGTCG |
| MrkH Box mut-5 (for) | TGGATCCGCGTTTTCATCTCCCAATGACTGTTTATTAATAGTCG |
| MrkH Box mut-6 (for) | TGGATCCGCGTTTTCATCTAAGAATGACTGTTTATTAATAGTCG |
| MrkH Box mut-7 (for) | TGGATCCGCGTTTTCATCTATCTTTGACTGTTTATTAATAGTCG |
| MrkH Box mut-8 (for) | TGGATCCGCGTTTTCATCTATCAAACACTGTTTATTAATAGTCG |
| UP mutation (for) | CATCTATCAATGACTGTTGCAGCATAGTCGATGGTTATCTG |
| UP mutation (rev) | CAGATAACCATCGACTATGCTGCAACAGTCATTGATAGATG |
| -84 to +166 (BamHI) | AGGATCCATCTATCAATGACTGTTTATTAATAGTCG |
| -77 to +166 (BamHI) | AGGATCCAATGACTGTTTATTAATAGTCGATGGTTATCTG |
| -71 to +166 (BamHI) | AGGATCCTGTTTATTAATAGTCGATGGTTATCTG |
| -67 to +166 (BamHI) | AGGATCCTTATTAATAGTCGATGGTTATCTGTTATATAAC |
| mrkH(NdeI)11a | ACATATGACAGAGGGAACGATAAAGACC |
| mrkH(BamHI)11a | AGGATCCTTAGTGGTGGTGGTGGTGGTGGTGGTGGATTCTCTTTTTGCGCTTGGCTTC |
| MrkH(SalI)F | TACGTCGACACCGCTACTGGCACTATAGA |
| MrkH(BamHI)R | TGGATCCGCGGAGCGCATTCAGCAGAT |
| MrkH15AS16F | GTATGAAATTATTGCTGCTTCTATTTTCAGAGAGGAG |
| MrkH15AS16R | CTCCTCTCTGAAAATAGAAGCAGCAATAATTTCATAC |
| MrkH39AS40F | CAGTATCATAACCCAAGCTTCTCTGACGCGCGTGGAC |
| MrkH39AS40R | GTCCACGCGCGTCAGAGAAGCTTGGGTTATGATACTG |
| MrkH202AS203F | GCTACTATCAGATAGCTTCTTCCTGCCAGTTTAAG |
| MrkH202AS203R | CTTAAACTGGCAGGAAGAAGCTATCTGATAGTAGC |
| MrkH217AS218F | GACCAGCGCAGAATAGCTTCTGAGAAGATACTGCTG |
| MrkH217AS218R | CAGCAGTATCTTCTCAGAAGCTATTCTGCGCTGGTC |
